# Synthesis and self-assembly behavior of a biodegradable and sustainable soybean oil-based copolymer nanomicelle

**DOI:** 10.1186/1556-276X-9-391

**Published:** 2014-08-12

**Authors:** Lixia Bao, Longchun Bian, Mimi Zhao, Jingxin Lei, Jiliang Wang

**Affiliations:** 1 State Key Laboratory of Polymer Materials Engineering, Sichuan University, Chengdu 610065, China; 2 School of Chemical Science and Technology, Yunnan University, Kunming 650091, China

**Keywords:** Soybean oil, Self-assembly, Nanomicelle, Biomaterials

## Abstract

Herein, we report a novel amphiphilic biodegradable and sustainable soybean oil-based copolymer (SBC) prepared by grafting hydrophilic and biocompatible hydroxyethyl acrylate (HEA) polymeric segments onto the natural hydrophobic soybean oil chains. FTIR, H^1^-NMR, and GPC measurements have been used to investigate the molecular structure of the obtained SBC macromolecules. Self-assembly behaviors of the prepared SBC in aqueous solution have also been extensively evaluated by fluorescence spectroscopy and transmission electron microscopy. The prepared SBC nanocarrier with the size range of 40 to 80 nm has a potential application in the biomedical field.

## Background

Many efforts have been done to develop biodegradable biomaterials during the past 2 decades due to their large potential application in biomedical fields of tissue engineering, gene therapy, regenerative medicine, controlled drug delivery, etc. [1-3]. There are many factors to choose biodegradable rather than biostable materials for biomedical applications. The main driving forces are the long-term biocompatibility issues with many of the existing permanent implants and many levels of ethical and technical issues associated with revision surgeries [4]. The recent research interest about biomaterials focuses on designation and development of novel biodegradable polymers and related derivates, including polyesters [5-7], polylactides [8], polycaprolactones [9-11], poly(ester amide)s [12,13], polyanhydrides [14-16], polyurethanes [17-20], and so on. Unfortunately, most of the reported main raw materials used to synthesize biodegradable polymers are unsustainable petroleum-based compounds. As the global demand for petroleum-based plastics continues to increase, unstable crude oil price and related environmental problems have triggered a search for replacing these non-biodegradable and unsustainable plastics. Development and application of biodegradable and sustainable plant-based products such as natural oils may be the most promising choice to solve these problems. For example, Thamae et al. [21] have developed a biodegradable corn stover filled polyethylene biomaterials. The effect of the corn stover size and the content and the morphology of the filler on the structure and mechanical properties of the obtained biocomposites have been extensively evaluated. Recently, our group has also developed a novel nontoxic, biodegradable, and ion-conductive plasticizer based on natural citric acid for soft poly(vinyl chloride) composites [22].

Soybean oil is one of the most widely available biodegradable and sustainable edible oils. From the angle of the chemical structure, soybean oil is a triglyceride with two dominant fatty acid residues, linoleic acid and oleic acid, and an average number of double bonds per molecule of 4.6. The average molecular weight of soybean oil is about 874, and it contains 51% of linoleic acid, 25% of oleic acid, 11% of palmitic acid, 9% of linolenic acid, and 4% of stearic acid residues [23]. The existence of the unsaturated double bonds in soybean oil molecules supplies opportunities for designing and modifying of soybean oil-based biodegradable polymers. Can et al. [24] have successfully prepared a rigid soybean oil-based thermosetting copolymer by a free radical copolymerization method. Biomaterials based on linseed oil monoglyceride maleates and modified acrylated epoxidized soybean oil with styrene have also been developed by Mosiewicki [25] and Colak [26], respectively. Recently, Cakmakli et al. [27] have reported the biocompatibility and the bacterial adhesion of a soybean oil-g-methyl methacrylate and butyl methacrylate copolymer for biomedical applications.

**Figure 1:**
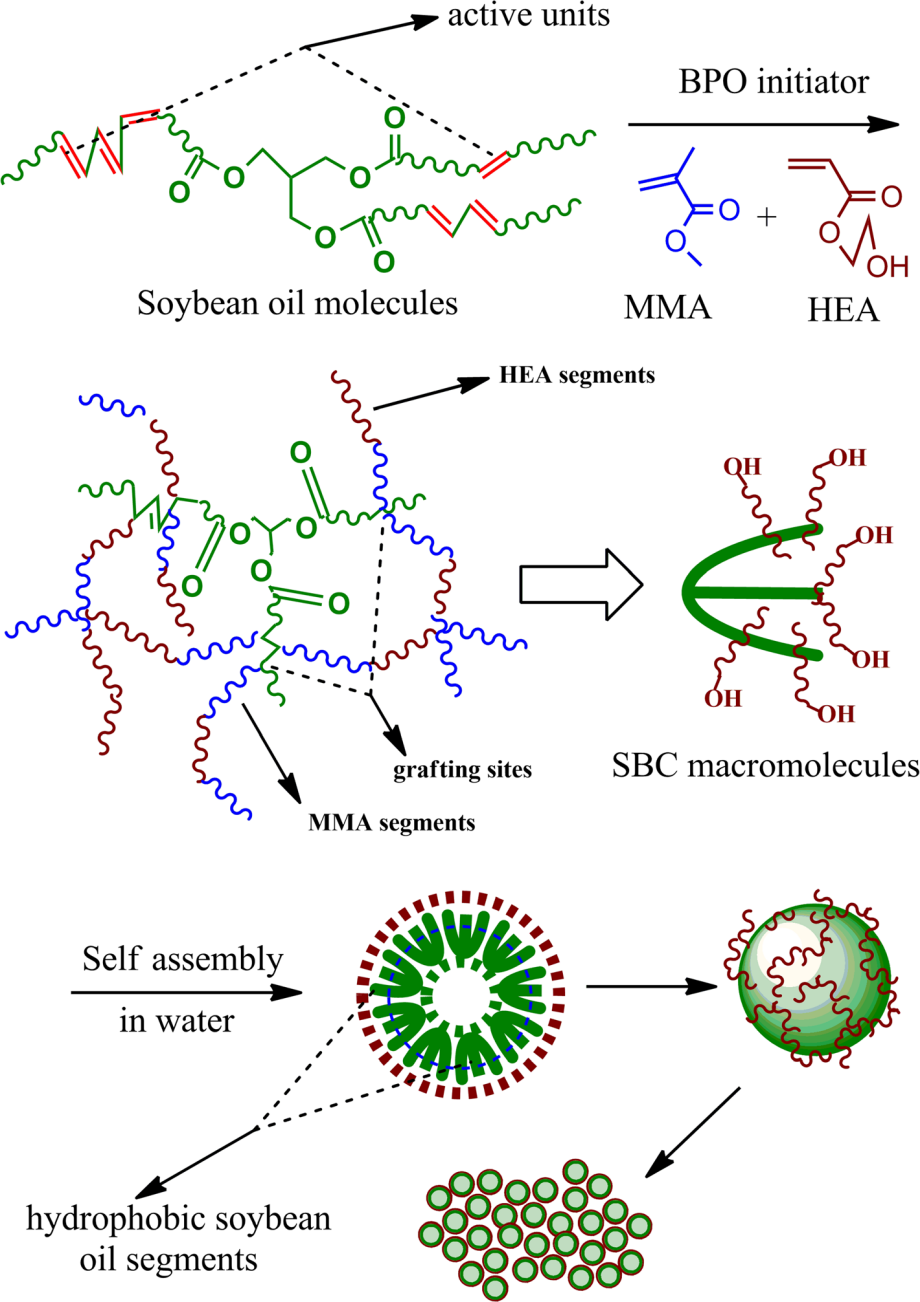
The synthesis route of the SBC macromolecules.

To the best of our knowledge, no studies have been conducted to develop amphiphilic nanoparticles for biomedicals (e.g., drug delivery) using soybean oil and its related copolymers. Recently, we have successfully prepared a novel monodispersed magnetic nanoparticle capped with oleic acid (including unsaturated double bonds) and acrylate copolymers [28]. In this work, we first report the self-assembly behaviors and the morphology of a novel amphiphilic biomacromolecule prepared by grafting biocompatible and non-toxic hydroxyethyl acrylate (HEA) hydrophilic segments onto the hydrobic soybean oil molecules. The synthesis route of the amphiphilic biomacromolecule is shown in Figure 1.

## Methods

### Synthesis of the soybean oil-based copolymer

The soybean oil-based copolymer (SBC) was prepared by a two-step batch grafting polymerization due to the fact that batch polymerization was usually facilitated to eliminate the heat of the polymerization and obtain polymers with uniform properties. In this procedure, 60 g soybean oil, 1 g methyl methacrylate (MMA), 2.5 g butyl acrylate (BA), 0.5 g hydroxyethyl acrylate (HEA), 1 g benzoyl peroxide (BPO), and 15 g ethyl acetate (EA) were first added into a flask with stirring at 75°C. The grafting polymerization reaction was maintained for 30 min. Four grams of BPO was quickly added into a mixed solution composed of 9 g MMA, 22.5 g BA, 4.5 g HEA, and 5 g EA. The mixture was then added into the flask dropwise for 3 h, and the reaction was maintained at 75°C for 7 h. The resulting SBC solution was then poured into hexane under stirring to remove unreacted soybean oil molecules, acrylate monomers, and related oligomers. The obtained SBC slurry was further dissolved into chloroform to get a solution with the SBC concentration of 50 mg/mL. Methanol was then added into the solution dropwise to further purify the grafted SBC macromolecules taking account of the different solubilities of SBC in chloroform and methanol. The obtained precipitation was dried under vacuum at 60°C overnight, and the target SBC was obtained.

### Self-assembly of the SBC in aqueous solution

To investigate the self-assembly behaviors and the morphology of the prepared SBC and the SBC nanomicelles, the purified SBC macromolecules were self-assembled in water and the corresponding procedures were listed as below. The SBC (1 wt.%) were first dissolved into dimethylacetamide (DMAc). Subsequently, deionized water was added dropwise under ultrasonification to avoid the precipitation of the SBC, and a 2 mg/mL SBC emulsion was obtained. The resulting emulsion was then transferred to dialysis tubes (MWCO-3500) and dialyzed against deionized water for 3 days to thoroughly remove the used DMAc. The obtained emulsion was further diluted by deionized water to yield a series of sample solution varying in the SBC concentration from 10^-4^ to 1 mg/mL.

### Characterizations

Un-polymerized soybean oil and the synthesized SBC were characterized by using a Nicolet-560 FTIR spectrometer with a resolution setting of 4 cm^-1^. The scanning range was altered from 400 to 4,000 cm^-1^. H^1^-NMR (400 MHz) spectrum of both soybean oil and the SBC was recorded on a Bruker AV-II spectrometer, using tetramethylsilane (TMS) as an internal standard in DMSO-d_6_ and CDCl_3_ as the solvent. Gel permeation chromatography (GPC) test of the synthesized SBC was performed by using an HLC-8320 GPC (Japan) at 25°C. Tetrahydrofuran and polystyrene with a narrow molecular weight distribution were used as the eluent and the reference, respectively. The flow speed of the solution was 1 mL/min. Steady-state fluorescence spectra of the SBC micelles were obtained using an F-7000 spectrophotometer (Hitachi, Tokyo, Japan) with a bandwidth of 2.5 nm and λ_em_ of 373 nm. Pyrene was used as the probe, and the final pyrene concentration was about 5 × 10^-7^ M. The morphology of the prepared SBC micelles was observed using a JEOL JEM-2100 electron microscope (TEM, JEOL Ltd., Tokyo, Japan) operating at an accelerating voltage of 200 kV.

## Results and discussion

**Figure 2:**
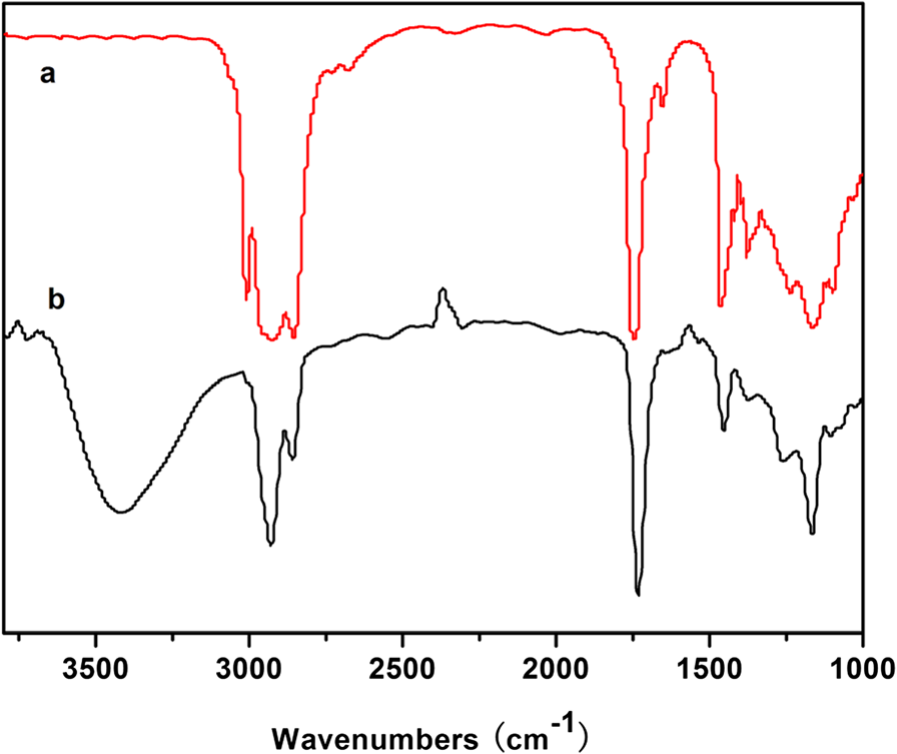
Spectrum of (a) FTIR of soybean oil and (b) FTIR of synthesized SBC.

Figure 2 (a, b) shows the FTIR spectra of pure soybean oil and the purified SBC, respectively. As can be seen from Figure 2 (a), obvious characteristic peaks at around 2,962, 2,923, 2,853, 1,463, and 1,455 cm^-1^ corresponding to -CH_3_ and -CH_2_ stretching vibrations are detected. In addition, characteristic peaks at about 3,008, 1,651, 1,746, and 1,099 cm^-1^ deriving from CH = CH, -C = C-, -COOC-, and -C-O-C- groups are also observed, indicating the existence of unsaturated double bonds in the soybean oil molecules. In the case of Figure 2 (b), apparent peaks similar with those of pure soybean oil at around 2,962, 2,928, 2,859, and 1,453 cm^-1^ corresponding to -CH_3_ and -CH_2_ stretching vibrations are detected. While characteristic peaks of -COOC- and -C-O-C- are found to shift from 1,746 and 1,099 to 1,732 and 1,106 cm^-1^ after the grafting polymerization. In addition, characteristic peaks at 3,008 and 1,651 cm^-1^ corresponding to CH = CH and -C = C- groups are not detected, showing that the unsaturated double bonds in soybean oil molecules can be successfully grafted by the selected monomers (i.e., acrylates). Moreover, characteristic peak at about 3,472 cm^-1^ deriving from the -OH stretching vibration of HEA is also observed, which is also an evidence to prove the grafting polymerization of soybean oil molecules.

**Figure 3:**
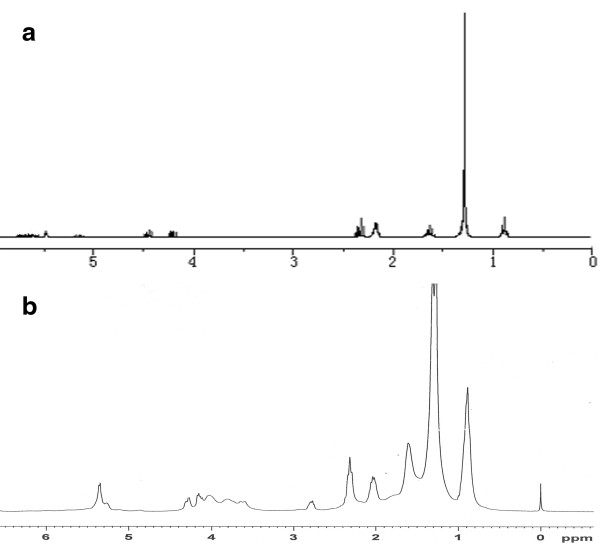
**H**^**1**^**-NMR of (a) soybean oil and (b) the synthesized SBC.**

Figure 3a, b shows the original H^1^-HMR spectra of pure soybean oil and the prepared SBC, respectively. As is shown in Figure 3b, characteristic peaks at around δ = 2.4, 2.2, 1.7, 1.3, and 0.9 ppm corresponding to the -CH_2_- group of unpolymerized soybean oil molecules (Figure 3a) are detected. In addition, the peaks at 5.2 and 4.0 to 4.3 ppm originating from the protons in the methyne and methylene groups of the triglyceride in soybean oil molecules are also observed, revealing the existence of the soybean oil segments in the SBC. Moreover, it is shown in Figure 3b that characteristic peaks at about 3.5 to 4.0 ppm deriving from the grafting segments (i.e., MMA-HEA-BA copolymers) are observed, which cannot be detected in the spectrum of soybean oil molecules (see Figure 3a). Characteristic peaks at about δ = 2.0 and 2.1 ppm corresponding to the grafting points have also been detected. H^1^-NMR results further indicate that acrylate copolymeric segments can be formed on the soybean oil molecules by the grafting polymerization.

Molecular information is very important for biomedical polymers, polymer with an over high molecular weight usually shows dramatic chain folds and entanglements, which will directly bring negative effects during the self-assembly process of the amphiphilic biomacromolecules. As can be seen from Table 1, the average molecular weight of the prepared SBC is 21, 369, which is similar with those of typical macromolecules for biomedical nanocarriers [29].

**Table 1:** GPC results of the prepared SBC

**Sample**	***M***_***w ***_**(g mol**^**-1**^**)**	***D *** **(*M***_***w***_ ***/M***_***n***_**)**
SBC	21, 369	3.2

**Figure 4:**
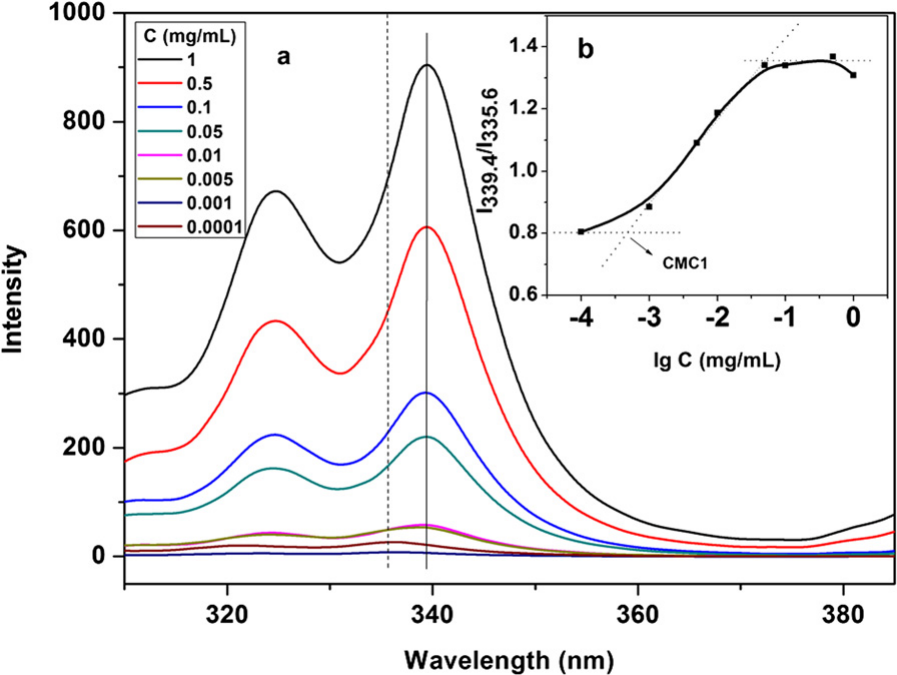
**Excitation spectra of different SBC micelles (a); influence of SBC concentration on ratio of I**_**339.4**_**/I**_**335.6 **_**(b).**

**Figure 5:**
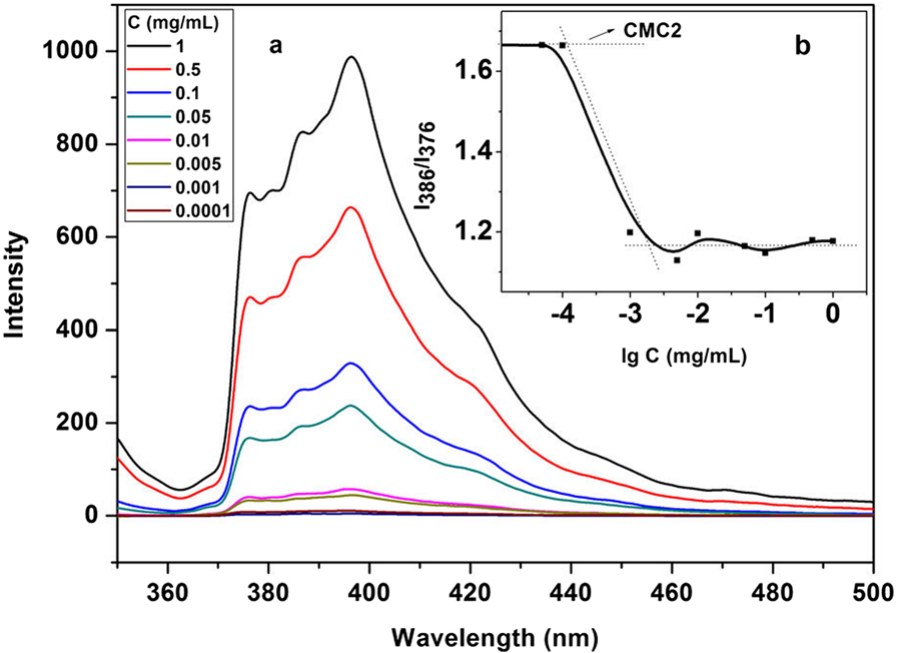
**Emission spectra of different SBC micelles (a); influence of SBC concentration on ratio of I**_**386**_**/I**_**376 **_**(b).**

It is well-known that amphiphilic macromolecules in a selective solvent can self-assemble into micelles containing dense cores of insoluble segments and outer shells formed by soluble segments. To confirm the formation of the SBC micelles in aqueous solution, a fluorescence technique has been used with a typical probe of pyrene [29], and the corresponding excitation and emission spectra are shown in Figures 4 and 5, respectively. It is shown in Figure 4a that the fluorescent intensity of the sample gradually increases from about 0 to 900 with ranging the SBC concentration from 10^-4^ to 1 mg/mL. The absorption band of the sample with a SBC concentration of 10^-4^ mg/mL has shifted from 335.6 to 339.4 nm when the SBC concentration reaches 1 mg/mL. As is shown in Figure 5a, the fluorescent intensity of characteristic peaks at about 376 and 386 nm also gradually enhance from around (0, 0) to (700, 900) with increasing the SBC concentration from 10^-4^ to 1 mg/mL. The above phenomena indicate that insoluble pyrene molecules have been gradually transferred from water to the inside of the SBC micelles with increasing the SBC concentration in aqueous solution [30-32].

Critical micelle concentration (CMC) is an important parameter to characterize the thermodynamic stability of micellar system upon dilution in nanomicelles *in vivo*. The ratio of I_339.4_/I_335.6_ in the excitation spectra is usually used to determine the CMC of amphiphilic molecules [30]. The influence of the SBC concentration in aqueous solution on the ratio of I_339.4_/I_335.6_ is shown in Figure 4b. The ratio of I_339.4_/I_335.6_ is found to dramatically increase from 0.8 to 1.38 with the enhancement of the SBC concentration from 1 × 10^-4^ to 4.9 × 10^-2^ mg/mL. It is almost unchanged with further increasing the SBC concentration from 4.9 × 10^-2^ to 1 mg/mL. Consequently, a CMC value of 4.57 × 10^-4^ mg/mL can be obtained from the intersection of the two tangent lines shown in Figure 4b.

Similarly, a typical ratio of I_3_/I_1_ (about I_383_/I_373_) of pyrene probe in emission spectra is also usually used to determine the CMC value of micelles. It is shown in Figure 5b, the ratio of I_3_/I_1_ rapidly decreases from 1.67 to 1.21 when the SBC concentration increases from 1 × 10^-4^ to 1 × 10^-3^ mg/mL. It only fluctuates near 1.18 with further increasing the SBC concentration from 1 × 10^-3^ to 1 mg/mL, revealing the un-sensitivity of the I_3_/I_1_ ratio at high SBC concentrations. A CMC value of 1.23 × 10^-4^ mg/mL (CMC_2_) can be also obtained from Figure 5b, which is slightly lower than the CMC_1_ observed from the excitation spectra. Consequently, the CMC value of the prepared SBC micelles is ranged from 1.23 × 10^-4^ to 4.57 × 10^-4^ mg/mL. The detected CMC value is much lower than those reported for well-known linear and nonlinear block copolymers, such as 4.1 × 10^-2^, 6.46 × 10^-2^, and 1.2 × 10^-3^ for conventional biodegradable thermogelling poly(ethylene glycol)/poly(ϵ-caprolactone) (PEG/PCL) diblock [33], branched PCL/PEG copolymers [34], and PCL/PEG/PCL triblock [35], respectively. It is as well lower than that (8.5 × 10^-4^ mg/mL) of recent reported biodegradable polyurethane micelles developed in our institute [29]. Such a low CMC value reveals that there is a strong tendency of the SBC molecules toward micelle formation in water, attributing to the good flexibility and the extraordinary surfactant features of the prepared SBC macromolecules. The low CMC value also indicates that the SBC micelles are highly thermodynamic stable, and that both the size and the polydispersity index of the SBC micelles are little changed with dilution [29].

**Figure 6:**
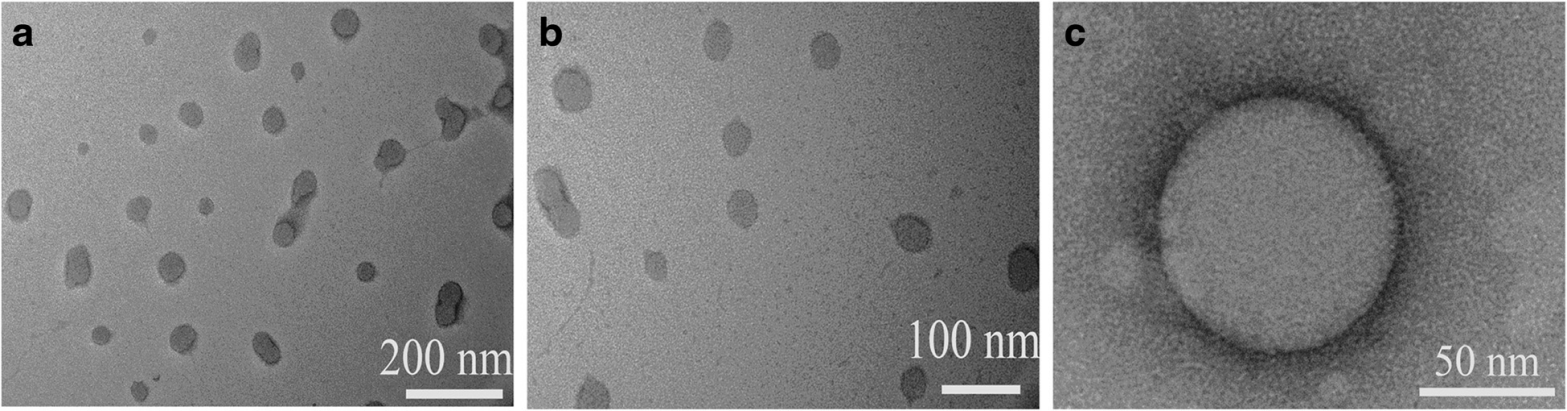
**TEM images of the SBC micelles at different magnifications (a, b, c).** The SBC concentration is 5 × 10^-3^ mg/mL.

TEM is a more powerful direct technique to investigate the formation of micelles. As is shown in Figure 6a, b, many spherical gray core and dark shell particles with a size range of 40 ~ 80 nm are found to evenly disperse in the view of TEM images. Meanwhile, a few double-bell-like nanoparticles (capsules) deriving from the aggregation of two neighbor particles are also detected, indicating that the number of nucleation centers of the SBC micellar solution with the concentration of 5 × 10^-3^ mg/mL is not enough to form uniform monodispersed micelles with a small particle size (such as 50 nm). In addition, Figure 6b also shows that the particle size distribution of the SBC micelles approaches 1.4, implying a semi-monodispersity of the prepared SBC nano-carriers in aqueous solution. To further investigate the spatial structure and the microenvironment of the SBC micelles, high-resolution TEM technique for a special selected SBC micelle has been used, and the corresponding TEM image is shown in Figure 6c. A clear and regular spherical nanoparticle composed of a gray core and a dark shell is obviously detected. The size of the observed SBC nanoparticle is near 72 nm. Moreover, by careful observation, one can see that the thickness of the shell layer of the observed SBC nanoparticle is about 7 nm, which should be the thickness of the monolayer self-assembled by the SBC macromolecules (see Figure 1). A few linear SBC aggregates (un-spherical) with the similar layer thickness are also detected in Figure 6a, b, which is also the evidence of self-assembly of the SBC macromolecules.

## Conclusions

In summary, a new biodegradable and nontoxic nanocarrier for potential drug delivery has been successfully prepared by grafting hydrophilic HEA polymeric segments onto the natural hydrophobic soybean chains. Fluorescence spectra studies show that the prepared SBC macromolecules can easily self-assemble to form core-shell nanoparticles in aqueous solution, and that the CMC of the prepared SBC is only 4.57 × 10^-4^ mg/mL, which is much lower than those of well-known biodegradable biomedical nanocarriers. TEM results indicate that the prepared SBC micelles are composed of a large amount of nanocarriers with the size range of 40 to 80 nm, and that the thickness of the SBC macromolecular monolayer each nanocarrier is about 1/10 of the diameter of the detected SBC micelle.

## Competing interests

The authors declare that they have no competing interests.

## Authors’ contributions

LXB, LCB, and ZMM carried out the preparation and main characterization of different samples and drafted the manuscript. JLW and JXL participated in the design of the study and the manuscript modification. All authors read and approved the final manuscript.
